# Diagnostic value of lung function tests in long COVID: analysis of positive bronchial provocation test outcomes

**DOI:** 10.3389/fmed.2024.1512658

**Published:** 2025-01-03

**Authors:** Wei Liu, Qixuan Feng, Xuefeng Yuan, Chang Lu, Shuang Wang, Yadong Yuan

**Affiliations:** ^1^First Department of Pulmonary and Critical Care Medicine, Hebei Chest Hospital, Shijiazhuang, China; ^2^Second Department of Pulmonary and Critical Care Medicine, The Second Hospital of Hebei Medical University, Shijiazhuang, China

**Keywords:** long COVID, lung, function test, diffusing capacity for carbon monoxide, bronchial provocation tests, diagnosis

## Abstract

**Background:**

Long COVID patients are prone to bronchial hyperresponsiveness and respiratory symptoms like coughing and breathing difficulties, often with positive bronchial provocation test (BPT) results.

**Objective:**

This study aims to evaluate the diagnostic value of various lung function tests in patients with long-term COVID-19, explicitly focusing on positive BPT outcomes.

**Methods:**

Our study analyzed the BPT outcomes and various pulmonary function parameters of all 9,406 COVID-19 patients who met the inclusion criteria and visited our hospital between February 24, 2022, and April 28, 2024. Key indicators included forced vital capacity (FVC), forced expiratory volume in one second (FEV1), peak expiratory flow (PEF), and single-breath diffusing capacity for carbon monoxide (DLCOc SB). A logistic regression model was employed to identify factors influencing positive BPT results, while the receiver operating characteristic (ROC) curve was used to assess the diagnostic efficacy of these indicators.

**Results:**

A total of 4211 valid samples were analyzed, with 3388 patients (80.46%) testing positive for BPT. Significant differences were observed between positive and negative groups regarding age, gender, smoking status (all *P* < 0.05), and specific lung function indicators, including FVC, FEV1/FVC ratio, maximum of vital capacity (VC max), and DLCOc SB (all *P* < 0.001). Logistic regression identified age, MEF50, and DLCOc SB as independent factors influencing positive BPT results. The area under the ROC curve for all assessed factors was <0.700, indicating limited diagnostic value.

**Conclusion:**

Age, the small airway function indicator MEF50, and the pulmonary diffusion function indicator DLCOc SB are independent influencing factors for BPT positivity in long-term COVID patients. However, baseline data and lung function indicators have limited utility for diagnosing positive BPT in this population, highlighting the complex nature of post-COVID respiratory symptoms.

## 1 Introduction

As patients gradually recover from coronavirus disease 2019 (COVID-19) infection, more studies show that the virus has long-term effects on multiple organs and systems in patients who have recovered from the acute phase (1–3). These effects are now commonly referred to internationally as the long-term impacts of COVID-19 or “Long COVID” syndrome. A retrospective cohort study in the United States assessed the risk of clinical sequelae after the acute phase of COVID-19 infection (4). The study reported that compared to adults who were not diagnosed with COVID-19 during the same period, there was an occurrence of at least one new respiratory, cardiovascular, hematologic, or neurological sequela after COVID-19 infection. Furthermore, respiratory failure due to fibrosis, interstitial thickening, and vascular abnormalities may persist 12 months after acute COVID-19 infection. Despite the gradual improvement in lung physiology and exercise capacity in COVID-19 patients post-infection, persistent physiological and imaging abnormalities may still be present 12 months after discharge.

The short-term and potential long-term sequelae of COVID-19 infection can significantly impact lung function. Among the various types of respiratory diseases caused by COVID-19 infection, lung function damage occurs to varying degrees. Dyspnea is one of the common symptoms following COVID-19 infection. Follow-up studies on respiratory symptoms 1–12 months after hospital discharge in COVID-19 patients have shown that persistent dyspnea in hospitalized patients ranges from 5 to 81% (5–7). Some individuals experience persistent Long COVID symptoms, manifesting as chronic cough and irritant cough.

Long COVID patients may experience long-term respiratory effects, such as airway inflammation, reduced lung function, or airway hyperresponsiveness. Studies have shown that the positive rate of bronchial provocation tests (BPT) is significantly increased in COVID-19-positive patients (8, 9). The BPT involves using chemical, physical, or biological stimuli to induce airway contraction, causing bronchial smooth muscle contraction, followed by lung function tests to determine the degree of bronchial narrowing. This test is a method used to measure airway hyperresponsiveness (AHR). It is one of the practical diagnostic standards for detecting bronchial asthma, asthmatic bronchitis, and allergies (10). However, the increased positive rate of BPT does not mean these patients can be diagnosed with asthma. Although respiratory symptoms caused by COVID-19 infection may resemble asthma, their pathological mechanisms are significantly different (11). Therefore, a comprehensive judgment needs to be made by combining the patient’s medical history and laboratory test results. Lung function tests can objectively assess a patient’s lung capacity, airflow limitation, and overall lung performance, while BPT detects airway sensitivity and potential asthma-like responses. These tests help to understand the respiratory impact of long-term COVID-19 and provide scientific evidence for developing more targeted treatment plans for patients.

This study analyzes the baseline data and various lung function indicators of COVID-19 patients who sought medical care and exhibited long-term respiratory symptoms post-infection. By collecting and examining these data, we intend to evaluate the impact of each indicator on the positive outcomes of BPT and provide further insights into the study of long-term COVID-19.

## 2 Materials and methods

### 2.1 Experimental design

This study is a retrospective analysis of lung function indicators in patients with long COVID. We categorized patients into positive and negative groups based on the results of BPT. The collection period for all COVID-19-infected patients was from February 24, 2022, to April 28, 2024. The study received approval from ethics committee of Hebei Chest Hospital (2020-R016), which conducted the research. Since this is a retrospective study, informed consent from patients was not required. This study included all COVID-19 patients who visited our hospital within the data collection period and met the inclusion and exclusion criteria.

### 2.2 Operational definition

The definition of “Long COVID” is based on the World Health Organization’s definition, which describes it as occurring in individuals with a confirmed or suspected history of COVID-19 infection, typically occurring within 3 months of the onset of COVID-19, with symptoms and effects lasting for at least 2 months and cannot be explained by the diagnosis of another disease (12). Additionally, it aligns with the definition provided by the Centers for Disease Control and Prevention (CDC), which states that at least 4 weeks after SARS-CoV-2 infection, no replicable virus can be detected, but patients continue to experience persistent symptoms or health problems (13).

### 2.3 Inclusion and exclusion criteria

Inclusion criteria: (1) History of COVID-19 infection; (2) Meeting the definitions of Long COVID provided by the World Health Organization and CDC; (3) Age ≥ 18 years; (4) Patients presenting with various respiratory and pulmonary symptoms primarily characterized by recurrent chest tightness and dyspnea.

Exclusion criteria: (1) Recent reinfection with the COVID-19 virus; (2) Patients with a documented allergic reaction to inhaled provocation agents or other parasympathetic drugs; (3) Patients with a history of fatal asthma attacks, those requiring mechanical ventilation for asthma exacerbation in the past 3 months, or those with severe chronic obstructive pulmonary disease (COPD) or interstitial lung disease; (4) Individuals who experienced myocardial infarction or other severe cardiovascular diseases within the past 3 months; (5) Severe respiratory diseases such as pulmonary embolism; (6) Individuals who have undergone recent chest surgery or experienced chest trauma; (7) Patients with other severe diseases affecting organs or tissues, and pregnant women.

### 2.4 Steps of the BPT detection

The inhalation BPT is the most commonly used method for clinically assessing airway hyperresponsiveness. Professional technicians from the pulmonary function department of this hospital performed the BPT. In the BPT detection, the tools used are standardized pulmonary function testing equipment (MasterScreen™ PFT System, Jaeger, Germany) and structured record forms to document the patient’s responses, test results, and relevant clinical data. The detailed examination process includes the following steps: (1) The subject adopts a sitting or supine position, uses a nose clip, and holds a mouthpiece. Then, the patients perform the breathing actions as instructed by the physician to assess baseline lung function. (2) The subject inhales saline as directed by the physician, followed by repeated lung function testing. (3) The subject pinches their nose and calmly inhales the provocation agent (acetylcholine chloride diluted with saline at concentrations ranging from 0.03 to 16 mg/ml, increasing in doubling dilutions), starting from the lowest concentration and gradually increasing the dose. Lung function tests are repeated after inhalation until FEV1 decreases by 20% or more from the baseline value, the highest concentration is reached, or the subject experiences significant discomfort. (4) If the BPT is positive and is accompanied by significant shortness of breath or wheezing, a bronchodilator should be administered to alleviate the subject’s symptoms. The test should be terminated after 10–20 min when lung function indicators return to baseline. A PC20 FEV1 of 8 mg/ml or a PD20 FEV1 of 12.8 μmol indicates a positive test, while values greater than these indicate a negative test. The pulmonary function instrument is used to record the patient’s respiratory function response. All data should be collected through standardized procedures to ensure the consistency and comparability of the measurements.

### 2.5 Testing indicators

We summarized and organized the baseline data of the included patients, including age, gender, occupation, and BMI (kg/m^2^). The lung ventilation testing indicators include Forced Vital Capacity (FVC), Vital Capacity Max (VC MAX), Forced Expiratory Volume in One Second (FEV1), FEV1/FVC ratio, Peak Expiratory Flow (PEF), Maximum Expiratory Flow (MEF), and Maximum Voluntary Ventilation (MVV). FVC measures the exhaled air volume after a maximal inhalation and is a key indicator for assessing lung capacity and airway patency. VC MAX represents the maximum volume of air a person can exhale after taking a deep breath, reflecting the overall lung volume. FEV1 quantifies the air exhaled in the first second of forced exhalation and is used to assess the degree of airway obstruction. The FEV1/FVC ratio helps differentiate between obstructive and restrictive lung diseases; a reduced ratio suggests airway obstruction, commonly seen in conditions like COPD and asthma. PEF is the highest flow rate during forced exhalation and reflects the airway’s ability to expel air. MEF measures the maximum expiratory flow at different lung volumes, such as MEF25, MEF50, and MEF75, which evaluate airflow in small, medium, and large airways. MVV is the maximum amount of air a person can breathe in and out in a minute under voluntary effort and assesses the overall ventilatory capacity of the lungs.

In addition to lung ventilation, lung diffusion function is assessed by indicators such as Single-Breath diffusing capacity for carbon monoxide (DLCOc SB) and DLCOc/VA (the ratio of DLCO to alveolar volume). DLCOc SB measures how efficiently carbon monoxide diffuses across the alveolar-capillary membrane into the bloodstream and helps detect conditions that impair gas exchange, such as interstitial lung diseases and pulmonary fibrosis. DLCOc/VA is the ratio of DLCO to alveolar volume, helping to identify the cause of gas exchange abnormalities. A reduced ratio may indicate issues such as pulmonary fibrosis or vascular abnormalities. In contrast, a standard or high ratio could suggest a primary decrease in lung volume without gas exchange issues. These indicators are essential for assessing the functionality of the lungs’ ventilation and diffusion systems and provide critical information for diagnosing and managing pulmonary diseases.

### 2.6 Statistical methods

This study’s data organization and analysis were performed using SPSS version 25.0. To ensure data quality, we employed standardized data collection tools and systems, which were carefully designed and validated to maintain accuracy and consistency throughout the study. The tool’s psychometric properties, such as reliability (Cronbach’s α) and validity [Kaiser-Meyer-Olkin (KMO) Test and Bartlett’s Test of Sphericity], were rigorously assessed. Reliability was tested through repeated measurements to ensure consistency in the data. In contrast, validity was ensured by confirming that the tool accurately measured the intended variables, as reflected in established clinical guidelines. Furthermore, all personnel involved in data collection were trained to use the tools appropriately, and data entry was closely monitored for errors to maintain high data quality.

Categorical data are expressed as counts and percentages, with statistical analysis conducted using the chi-square test. Continuous data are presented as mean ± standard deviation (normally distributed data) or median (25%, 75%) (non-normally distributed data). The t-test is used to compare continuous data between two normally distributed groups. Non-parametric tests are used to compare two groups that are not normally distributed. Collinearity diagnostics are performed to analyze the collinearity between different factors. A variance inflation factor (VIF) of <5 indicates high collinearity between factors, while a VIF of ≥5 indicates significant collinearity; in this case, factors that are relatively unimportant and have high VIFs are excluded based on clinical experience. A logistic regression analysis model using a meticulous two-step method is employed to calculate the diagnostic significance of each factor on the outcomes. ROC curves are used to evaluate the diagnostic efficacy of the indicators included in the regression analysis. In this study, a *P*-value of <0.05 is considered statistically significant.

## 3 Results

### 3.1 Baseline information of patients

The study population consisted of all patients who had been infected with COVID-19 and sought treatment at Hebei Chest Hospital for Long COVID. Out of a total sample of 9,406 patients who had recovered from COVID-19, 4,110 valid samples were included in this study, with 3,388 patients (80.46%) testing positive for the BPT and 722 patients (19.54%) testing negative.

The median age of patients who tested positive for the BPT was 53.0 (39.0, 63.0), while the median age of negative patients was 59.0 (48.0, 69.0), showing a significant age difference between the two groups (*P* < 0.001). The gender comparison results indicated that the positive rate for men was 82.94% (1,415/1,706), while the positive rate for women was 86.57% (1,973/2,279), with a significant difference in gender distribution (*P* = 0.001); and the missing rate of patients’ gender information is 3.0% (125/4110).

There were also significant differences regarding smoking status and different occupations between the two groups (both *P* < 0.001). The smoking rate among BPT-positive patients is 24.5% (830/3388), while the BPT-negative patients is 32.7% (195/597); and the missing rate of patients’ smoking status information is 3.0%. There is no significant difference in BMI (kg/m^2^) between the two groups (*P* = 0.062) (Table 1).

**TABLE 1:** The statistical results of baseline data.

Factors	Groups	BPT positive (*n* = 3388)	BPT negative (*n* = 722)	X^2^/t/Z	*P*
Age (years)	–	53.0 (39.0, 63.0)	59.0 (48.0, 69.0)	9.437	<0.001
Gender	Male	1415	291	10.096	0.001
	Female	1973	306		
BMI (kg/m^2^)		24.6 (22.1, 27.3)	24.2 (22.0, 26.8)	1.863	0.062
Smoking	No	2558	402	17.711	<0.001
	Yes	830	195		
Occupation	Farmer	1618	299	36.440	<0.001
	Company employee	1080	140		
	Retired/unemployed	563	148		
	Students	118	10		
FEV1/FVC (%)		76.7 (70.6, 81.4)	78.1 (70.9, 82.2)	2.665	0.008
FEV1		100.6 (86.8, 111.8)	100.7 (82.8, 113.1)	0.513	0.608
VC MAX		106.1 (95.1, 117.1)	102.8 (88.2, 116.5)	4.181	<0.001
FVC		108.9 (97.6, 120.3)	105.7 (91.1, 119.7)	3.953	<0.001
FEV 1% FVC		91.5 (84.1, 97.0)	93.4 (85.1, 98.1)	3.550	<0.001
MEF 25		51.2 (33.2, 70.4)	53.2 (33.2, 76.1)	1.745	0.081
MEF 50		74.8 (52.9, 94.4)	80.3 (54.8, 104.3)	3.241	0.001
MEF 75		93.2 (72.1, 110.4)	98.2 (71.8, 114.8)	2.475	0.013
MMEF 75/25 (%)		66.4 (45.3, 85.8)	70.5 (45.3, 92.6)	2.635	0.008
MVV		96.0 (80.2, 111.8)	94.1 (74.8, 112.9)	1.037	0.300
DLCOc SB		89.2 (80.0, 101.2)	85.2 (65.5, 98.7)	5.049	<0.001
DLCOc/VA		93.9 (83.0, 104.8)	93.5 (79.6, 105.7)	1.525	0.127

BPT, bronchial provocation tests; BMI, Body mass index; FEV1, forced expiratory volume in 1 s; FVC, forced vital capacity; VC MAX, vital capacity max; FEV 1% FVC, FEV in 1%/FVC; PEF, peak expiratory flow; MEF, maximal expiratory flow; MEF 25/50/75, MEF at 25%, 50%, and 75%; MVV, maximum voluntary ventilation; DLCOc SB, the single-breath diffusing capacity for carbon monoxide; SB, single-breath; DLCOc/VA, DLCOc/alveolar volume; VA, alveolar volume. The categorical data between the two groups was compared using the chi-square test. If both groups followed a normal distribution, a t-test with P-value was used for continuous data. For at least one group not following a normal distribution, the Mann-Whitney U (non-parametric) test was applied, with results presented as Z and P values. A *P*-value of less than 0.05 indicated a significant difference between the two groups.

### 3.2 Detection of lung function indicators

The pulmonary ventilation indices showed that the FVC in patients with a positive bronchial provocation test (BPT) was higher than that in negative patients, with a statistically significant difference between the two groups (*Z* = 3.953, *P* < 0.001). A similar trend was observed in the comparison of VC MAX between the two groups (*Z* = 4.181, *P* < 0.001). However, in the comparisons of FEV1/FVC (%) (*Z* = 2.665, *P* = 0.008) and FEV1%FVC (*Z* = 3.550, *P* < 0.001), the positive group had lower values than the negative group. On the other hand, there was no statistically significant difference in FEV1 or MVV between the two groups (both *P* > 0.05) (Table 1).

Regarding the indices of maximal expiratory flow (including MEF25, MEF50, MEF75, and MMEF75/25), the results showed that the positive group had lower values for MEF50, MEF75, and MMEF75/25 compared to the negative group, with statistically significant differences (all *P* < 0.05). However, there was no significant difference between the two groups for MEF25 (*P* = 0.146) (Table 1).

For pulmonary diffusion function indices, the DLCOc SB values in the BPT-positive group were higher than those in the negative group, with a statistically significant difference (*P* < 0.001). However, no significant difference was observed in DLCOc/VA between the two groups (*P* = 0.127) (Table 1).

### 3.3 Establishment of the logistic regression analysis model

Collinearity diagnostic results indicated that there was multicollinearity between FEV1 and FEV1/FVC (%) (VIF > 5). Therefore, FEV1 was excluded from the logistic regression analysis model. The logistic regression analysis results showed that age, MEF 50, and DLCOc SB were independent factors associated with the positivity of the BPT (all *P* < 0.05). Other parameters, such as gender, smoking status, occupation, and other lung ventilation and diffusion function indicators, did not have an impact on the positivity of the BPT in COVID-19-infected patients (all *P* > 0.05) (Table 2).

**TABLE 2:** The results of the logistics analysis.

Factors	Groups	Univariate analysis		Multivariate analysis	
		**OR**	***P***	**OR**	***P***
Age (years)		0.974 (0.969, 0.979)	**<0.001**	0.979 (0.969, 0.988)	**<0.001**
Gender (M/F)		1.279 (1.089, 1.503)	**0.003**	1.007 (0.756, 1.343)	0.960
BMI (kg/m^2)		1.009 (0.988, 1.030)	0.392	–	–
Smoking		0.694 (0.583, 0.827)	**<0.001**	0.760 (0.559, 1.033)	0.079
Work	On job^a^	1.442 (1.183, 1.757)	**<0.001**	1.054 (0.770, 1.441)	0.745
	Retired/unemployed^a^	0.730 (0.595, 0.895)	**0.003**	0.868 (0.669, 1.126)	0.287
	Students^a^	1.901 (1.080, 3.347)	**0.026**	0.607 (0.240, 1.534)	0.291
FEV1/FVC		1.000 (1.000, 1.000)	0.379	–	–
VC MAX		1.007 (1.003, 1.010)	**0.002**	1.001 (0.996, 1.005)	0.810
FVC		1.006 (1.002, 1.010)	**0.003**	1.000 (0.995, 1.004)	0.876
FEV 1 % FVC		0.999 (0.996, 1.002)	0.632	–	–
MEF 25		0.997 (0.995, 1.000)	**0.023**	1.002 (0.995, 1.009)	0.590
MEF 50		0.999 (0.997, 1.000)	0.116	0.994 (0.988, 1.000)	**0.043**
MEF 75		0.999 (0.996, 1.001)	0.242	–	–
MMEF 75/25		0.998 (0.996, 1.000)	0.060	0.993 (0.984, 1.002)	0.147
MVV		1.000 (0.998, 1.003)	0.656	–	–
DLCOc SB		1.014 (1.010, 1.019)	**<0.001**	1.015 (1.008, 1.021)	**<0.001**
DLCOc/VA		1.007 (1.002, 1.013)	**0.008**	1.000 (0.997, 1.003)	0.879

^a^Compare and contrast with subgroup I. Bold value indicates *P* < 0.05.

### 3.4 Diagnostic value of various factors

**FIGURE 1:**
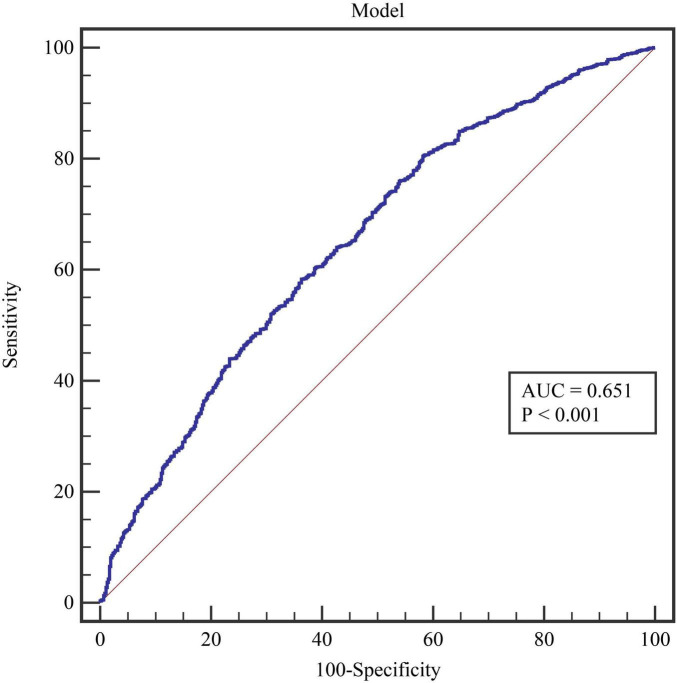
The results of the model ROC curve.

We evaluated the diagnostic value of 11 essential indicators for determining the positivity of the BPT. The results indicated that all factors had low diagnostic value (all AUC < 0.700) (Table 3). Among these, the indicator with the highest diagnostic value was the patient’s age (AUC = 0.612), which also had the highest diagnostic specificity (59.83%). The indicator with the highest diagnostic sensitivity was MEF 50 (84.33%). The model constructed using age, MEF 50, and DLCOc SB had an AUC of 0.651, with a sensitivity of 80.45% and a specificity of 41.83% (Figure 1).

**TABLE 3:** The results of the ROC curve.

Factor	AUC	SE	Sensitivity (%)	Specificity (%)	Youden index	AC	z-statistic	*P*
Age (years)	0.612	0.012	56.85	59.83	0.167	=55.0	9.676	<0.001
BMI	0.516	0.012	66.62	37.73	0.043	>23.1	1.367	0.172
Smoking	0.537	0.012	75.50	31.86	0.074	=0	3.901	<0.001
FEV1/FVC	0.532	0.012	58.05	47.78	0.058	=78.2	1.933	0.053
VC MAX	0.547	0.012	83.41	26.73	0.101	>89.9	3.801	<0.001
FEV 1 % FVC	0.533	0.012	58.32	49.23	0.075	=93.1	2.704	0.007
MEF 25	0.513	0.012	80.08	25.24	0.053	=75.6	1.043	0.297
MEF 50	0.529	0.012	84.33	23.82	0.082	=104	2.280	0.023
MMEF 75/25	0.522	0.012	82.42	24.27	0.067	=91.7	1.797	0.072
DLCOc SB	0.576	0.012	81.38	32.99	0.144	>75	4.914	<0.001
DLCOc/VA	0.530	0.012	75.68	32.29	0.080	>82.8	1.941	0.052
Model	0.651	0.012	80.45	41.83	0.223	>0.7	10.540	<0.001

ROC, receiver operating characteristic curve; AUC, area under the ROC curve; SE, standard error; AC, associated criterion.

### 3.5 Reliability and validity testing

This study’s reliability analysis of all 17 included indicators showed a Cronbach’s α value of 0.837. The content validity test yielded a KMO Test value of 0.853, and Bartlett’s Test of Sphericity showed a result of *P* < 0.001. These results indicate that the selected measurement indicators in this study are highly reliable.

## 4 Discussion

This study analyzed lung examination data from 4,211 long COVID patients who received diagnosis and treatment in our hospital. The results revealed that 80.46% of these patients tested positive for bronchial provocation tests. This indicates that COVID-19 infection can lead to long-term adverse effects on the human body, further corroborated by the findings of Guinto et al. (14, 15). In this study, the median age of patients who tested positive in the BPT was 53.0 (39.0, 63.0), which is slightly younger than the median age of 59.0 (48.0, 69.0) in the negative group, with a statistically significant difference between the two groups (*P* < 0.001). The age difference may be due to varying sensitivities to bronchial and airway hyperreactivity (BHR) across different age groups. Generally, children tend to exhibit higher bronchial hyperreactivity compared to adults, partly due to developmental differences in airway structure and function, making children’s airways more reactive to stimuli (16). In middle-aged and older populations, BHR typically declines with age. This decline may be related to changes in lung function and airway caliber, ultimately affecting the results of BPT in the elderly (17, 18). There was also a statistical difference in gender between the two groups (*P* = 0.001), with a BPT positivity rate of 82.90% in males and 86.67% in females. Research shows that females often exhibit higher sensitivity and reactivity in BPT than males, which may stem from differences in hormone levels between the two sexes (19). The incidence of asthma and airway hyperreactivity is typically higher in females than males, especially during puberty and adulthood. Studies have indicated that specific bronchial provocation agents (such as acetylcholine and histamine) also differ between genders, which is one reason for the higher positivity rate in females (20). Research indicates that smoking significantly enhances bronchial hyperreactivity, thereby affecting the results of BPT. The increased pulmonary reactivity in smokers is attributed to chronic inflammatory states induced by tobacco smoke, impairment of pulmonary defense mechanisms, and the inherent toxicity of cigarettes. These adverse factors persist in the smoking population, ultimately leading to a higher positivity rate in BPT among this group (21). The results of this study further support these points. However, in this study, the BMI values of the patient population did not affect the positivity of the BPT (*P* > 0.05).

In this study, various pulmonary function indicators, including FVC, FEV1/FVC (%), VC MAX, FEV1% FVC, MEF 50, 75, and MMEF 75/25, showed significant differences between the two groups (all *P* < 0.05); however, MEF 25 did not exhibit statistical significance between the two groups (*P* > 0.05). MEF25 reflects the expiratory flow in the early phase, and this indicator decreases when there is intrathoracic upper airway obstruction (22). Recent studies suggest that MEF 25 may not significantly influence the results of BPT in all cases (22, 23). Changes in MEF 25 may not always correlate closely with bronchial hyperreactivity or asthma diagnoses in specific populations. Organizations like the American Lung Association recommend focusing on overall pulmonary function parameters rather than specific indicators, including MEF 25, in BPT (24). MEF50 reflects the expiratory flow in the middle phase and decreases when airflow is limited or when there are small airway lesions (25). MEF75 reflects the expiratory flow in the late phase and decreases when airflow is limited or when small airway lesions are present (26). The changes in these flow indicators can help assess the characteristics of different types of ventilatory dysfunction. Minor airway dysfunction is considered if any two indicators are below 65%. MMEF75/25 represents the average expiratory flow rate during 25–75% of the subject’s vital capacity, with average values typically greater than 65% (27). In long-term COVID patients, the decrease in MEF 50 and MEF 75 (mid and late expiratory flow rates) in those with a positive BPT is usually related to narrowing and small airway dysfunction. The possible causes include inflammation and structural changes in the small airways, increased sensitivity of the airways to external stimuli, early damage to small airways during the initial infection, and residual lung injury following the viral infection (28). However, the comparison of severe/critical and mild/moderate COVID-19 patients conducted by Krzysztof Kłos et al. showed that the MEF 25–75 values in both groups did not exhibit significant changes at 3 months (29). In this study, MEF 50 was found to be an independent factor influencing the bronchial provocation test results in long COVID patients. Thus, MEF50, as an independent factor for bronchial provocation test positivity in long-term COVID patients, can assist doctors in predicting airway reactivity, guiding bronchodilators or anti-inflammatory medications, and improving patients’ long-term respiratory function.

DLCOc SB measures the ability of the lungs to transfer inhaled gases into the bloodstream. When assessing BPT results, DLCOc SB values can indicate the extent of lung function impairment, particularly in long-term COVID-19, asthma, or other respiratory diseases. A reduction in DLCOc SB may be associated with more severe airway obstruction and hyperreactivity, leading to positive bronchial provocation test results. In the results of this study, the DLCOc SB test results of patients in the BPT positive group were higher than those in the negative group. The result may be because DLCOc SB measures the ability of the lungs to diffuse gases into the bloodstream. If the BPT is positive, the patient’s airways may have inflammation or hyperreactivity, leading to airway narrowing (30). However, the diffusion capacity of the alveoli may still temporarily compensate by enhancing gas exchange to maintain oxygen and carbon dioxide transfer, which could result in an elevated DLCOc SB during the test (31). Although an increased DLCOc SB can indicate that the lungs still have strong gas exchange ability under certain conditions, this does not necessarily mean the patient’s overall lung function is normal, especially in COVID-19 patients with bronchial hyperreactivity and airway inflammation. The specific cause needs to be analyzed in relation to the patient’s clinical symptoms, other lung function indicators, and further examinations.

The diffusing capacity for carbon monoxide (DLCO), standardized to alveolar volume (VA) and expressed as DLCOc/VA, is crucial for assessing pulmonary gas exchange efficiency. In COVID-19 patients, particularly those with long-term COVID-19, abnormalities in DLCOc/VA are linked to impaired lung function and persistent respiratory symptoms. Studies have shown that COVID-19 patients typically exhibit reduced DLCO and a decreased DLCOc/VA ratio, indicating damage to the alveolar-capillary membrane. Long COVID patients with persistent symptoms such as dyspnea often demonstrate lower DLCOc/VA values, suggesting long-term lung injury and ongoing inflammation (32, 33). However, in this study, lung diffusion function testing revealed a statistically significant difference in DLCOc SB between the two groups (*P* < 0.001), while DLCOc/VA did not show a significant difference (*P* = 0.121). This discrepancy may arise from variations in the severity and duration of symptoms experienced by Long COVID patients. Some research findings indicate that lung function impairment, particularly a reduction in DLCO/VA, is associated with persistent symptoms such as dyspnea and decreased exercise tolerance in severe COVID-19 cases. This impairment can last several months post-infection (33, 34). However, the direct impact of these impairments on the results of BPT in COVID-19 patients may not be pronounced.

After excluding multicollinearity issues, the remaining indicators were included in the logistic regression model. The results indicated that age, MEF 50, and DLCOc SB were independent influencing factors for positive BPT in patients with Long COVID (all *P* < 0.05). However, in subsequent ROC curve analysis, it was found that the diagnostic value of all indicators was below 70%, including the diagnostic performance of the model (ROC = 0.651). These findings suggest that none of the indicators included in this study demonstrated excellent diagnostic value for predicting positive BPT. This outcome may be attributed to the complex pathogenic causes and mechanisms associated with COVID-19 infection. Using conventional and limited indicators to predict the positivity of BPT presents significant challenges. The multifactorial nature of Long COVID, involving various respiratory and systemic complications, necessitates a more comprehensive approach to accurately assess and diagnose bronchial hyperreactivity and related conditions in this patient population. Further studies incorporating a broader range of clinical, functional, and inflammatory markers may enhance our understanding and improve diagnostic accuracy for these patients.

## 5 Conclusion

This study collected and analyzed baseline data and pulmonary function indicators of patients with long-term respiratory symptoms following COVID-19 infection to investigate the impact of these indicators on the positivity of BPT. The results indicated that age, the small airway function indicator MEF50, and the pulmonary diffusion function indicator DLCOc SB are independent factors for BPT positivity in long-term COVID-19 patients. Due to the complexity of COVID-19 infection, baseline data, lung ventilation, and diffusion capacity indicators have limited diagnostic value for identifying BPT positivity in long-term COVID-19 patients. Therefore, when evaluating and managing long-term COVID-19 patients, a more comprehensive selection of diagnostic methods and relevant indicators is necessary to achieve lung function recovery and symptom improvement.

## Data Availability

The original contributions presented in this study are included in this article/supplementary material, further inquiries can be directed to the corresponding author.
